# Cereblon attenuates DNA damage-induced apoptosis by regulating the transcription-independent function of p53

**DOI:** 10.1038/s41419-019-1317-7

**Published:** 2019-01-25

**Authors:** Liang Zhou, Guoqiang Xu

**Affiliations:** Jiangsu Key Laboratory of Neuropsychiatric Diseases and College of Pharmaceutical Sciences, Jiangsu Key Laboratory of Preventive and Translational Medicine for Geriatric Diseases, Soochow University, Suzhou, Jiangsu 215123 China

## Abstract

Cereblon (CRBN) is the substrate receptor of the cullin 4-RING E3 ligase complex and has been employed for targeted protein degradation in the treatment of cancers. However, its normal physiological functions and molecular mechanism in the regulation of DNA damage response are largely unknown. Here we find that CRBN plays a protective role against DNA damage-induced apoptosis in cell lines and primary cells. Mechanistic studies demonstrate that although CRBN does not affect the ubiquitination and degradation of the tumor suppressor p53, it directly interacts with p53 and therefore, suppresses the interaction between p53 and anti-apoptotic regulators Bcl-2 and Bcl-X_L_. CRBN depletion enhances the interaction between p53 and Bcl-2/Bcl-X_L_, reduces mitochondrial membrane potential, increases the cleavage of caspase-3 and poly(ADP-ribose) polymerase 1, and thus promotes DNA damage-induced apoptosis in cell lines and primary cells upon etoposide treatment. Moreover, *Crbn* knockout mice exhibit increased mortality upon etoposide challenge. Taken together, our data elucidate a novel molecular mechanism by which CRBN inhibits DNA damage response in vitro and in vivo. This work extends our understanding of the broad spectrum of physiological roles for CRBN and may suggest its potential application in the treatment of DNA damage-associated diseases.

## Introduction

p53 is one of the most studied tumor suppressors, which is critical for the regulation of DNA damage-induced apoptosis^1–4^. As a transcription factor, nuclear p53 promotes the expression of pro-apoptotic genes, such as apoptosis regulator *BAX*^5^ and Bcl-2-binding component 3 *PUMA*^6^. Their increased protein products then initiate mitochondrial damage, reduce mitochondrial membrane potential (MMP), release cytochrome c, activate caspases, and lead to the cleavage of poly(ADP-ribose) polymerase 1 (PARP1) and apoptosis^4,7^. However, the nuclear localization signal deleted p53 can still promote apoptosis, indicating the important role of cytoplasm p53 in the regulation of apoptosis^8^. The transcription-independent pro-apoptotic functions of p53 are closely associated with Bcl-2 family members^9^, such as Bcl-2^10^, Bcl-X_L_^11^, and BAX^5,11,12^. The stable interaction between p53 and anti-apoptotic regulators Bcl-2 and Bcl-X_L_ is the important molecular mechanism underlying DNA damage-induced apoptosis^13,14^. p53 stably binds to Bcl-2 and Bcl-X_L_, affects mitochondrial outer membrane permeabilization, and counteracts the protective role of Bcl-2 and Bcl-X_L_ in the regulation of mitochondrion-mediated apoptosis^10,11^. Therefore, both the transcription-dependent and -independent functions of p53 are involved in the regulation of DNA damage-induced cell death.

Cullin 4-RING E3 ligases (CRL4s) are a group of protein complexes consisting of cullin-4A/B, damage-specific DNA-binding protein 1, RING finger protein, and multiple substrate receptors, which recognize a specific set of substrates for ubiquitination^15^. CRL4s monoubiquitinate histone H2A and regulate DNA damage repair, which is mediated by the substrate receptor DDB2^16^. The substrate receptor Cdt2 promotes CRL4-mediated monoubiquitination of proliferating cell nuclear antigen in response to DNA damage^17^. Cereblon (CRBN), acting as a substrate receptor of the CRL4 E3 ligase, regulates a variety of cellular processes through the enzymatic function of its associated E3 ligase^18–20^. CRBN promotes the ubiquitination of large-conductance calcium-activated potassium (BK_Ca_) channel^20^, chloride channel protein 1 (CLC-1)^21^, and presynaptic proteins^22^, and thus potentially regulates learning and memory^23,24^. The pharmacological intervention in animal models demonstrates that the learning and memory deficits in *Crbn* knockout (KO) mouse are affected by exaggerated AMPK activity, inhibition of mTORC1 signaling pathway^24^, and increased activity of BK_Ca_ channel^23^. CRBN also enhances the ubiquitination and degradation of c-Jun, reduces the activity of c-Jun associated transcription factors, and thus suppresses lipopolysaccharide-induced inflammatory response^25^. CRBN mediates immunomodulatory drug (IMiD)-induced death of multiple myeloma cells by promoting the ubiquitination-mediated degradation of two transcription factors IKZF1 and IKZF3^26,27^. This principle has been utilized for the development of proteolysis targeting chimera in drug discovery by targeting previously “undruggable” proteins^28,29^. Furthermore, CRBN exhibits non-enzymatic functions independent to its associated E3 ligase in multiple processes, such as on epigenetic regulation of potassium voltage-gated channel subfamily A member 3 (*Kcna3*)^30^, formation of sequestosome-1/p62 bodies^31^, and inhibition of ubiquitination of tumor necrosis factor receptor-associated factor 6 and TAK1-binding protein 2^32^ through its interaction with DNA or proteins. In addition, two CRBN mutants C391R (Cys to Arg mutation at position 391) and R419X (Arg to stop codon mutation at position 419) have been discovered to be the genetic causes for different degree of intellectual disability, indicating the important roles of CRBN in the regulation of neuronal functions^33–35^. Moreover, it has also been discovered that CRBN protects neuronal cells from death induced by pathogenic protein aggregates in neurodegenerative diseases^31^. However, it is unknown whether CRBN could regulate DNA damage in neuronal and non-neuronal cells.

In this work, through pharmacological intervention and genetic manipulation, we discovered that CRBN deficiency in cell lines, primary fibroblasts, and cortical neurons leads to their increased sensitivity to DNA-damaging reagents such as etoposide and cisplatin. Detailed studies at the molecular level revealed that CRBN protects the etoposide-induced cell death by altering the interaction between p53 and Bcl-2/Bcl-X_L_ through its competitive interaction with p53 but not through the enzymatic function of its associated E3 ligase. In addition, in vivo experiments showed that *Crbn* KO mice exhibit increased mortality rate upon etoposide challenge. This work indicates that CRBN could protect cells from DNA damage-induced apoptosis, which provides a novel understanding of physiological roles of CRBN in p53-mediated apoptosis.

## Results

### CRBN reduces DNA damage-induced apoptosis

**Fig. 1:**
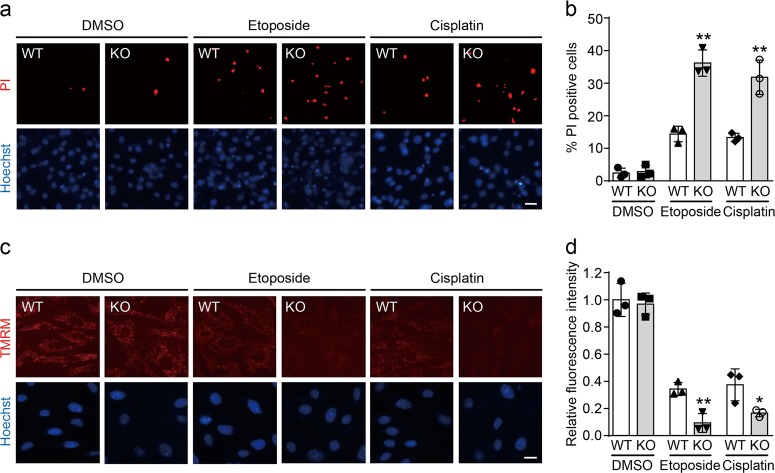
Cereblon (CRBN) deficiency decreases the viability of fibroblasts upon DNA damage. **a** CRBN depletion increases propidium iodide (PI)-positive primary fibroblasts upon etoposide or cisplatin treatment. Primary fibroblasts from wild-type (WT) and *Crbn* knockout (KO) littermate mice were exposed to etoposide (50 μM) or cisplatin (10 μM) for 48 h and then subjected to Hoechst and PI staining. Scale bar: 20 µm. **b** Quantitative data (mean ± SD) of **a** from three independent experiments. **P < 0.01, Student’s *t*-test. **c** CRBN deletion reduces the mitochondrial membrane potential of primary fibroblast cells upon etoposide or cisplatin treatment. Primary fibroblasts from WT and *Crbn* KO littermate mice were exposed to etoposide (50 μM) or cisplatin (10 μM) for 8 h and then subjected to tetramethylrhodamine methyl ester (TMRM) staining for microscope detection. Scale bar: 20 µm. **d** Quantitative data (mean ± SD) of **c** from three independent experiments. *P < 0.05, **P < 0.01, Student’s *t*-test

Although CRL4 E3 ligases regulate DNA damage response^36–40^, the role of one of its substrate receptors CRBN in DNA damage response is largely unknown. To explore this function, we first determine if CRBN affects apoptosis induced by DNA damage. We cultured the primary fibroblasts dissected from wild-type (WT) and *Crbn* KO littermate mice (Supplementary Figure S1) and then treated them with the DNA damage inducers etoposide and cisplatin. The immunofluorescence images of propidium iodide (PI) staining showed that *Crbn* KO fibroblasts are more susceptible to apoptosis induced by etoposide and cisplatin (Fig. 1a, b). Given the important role of mitochondria in DNA damage-induced apoptosis, we then examined the MMP in WT and *Crbn* KO fibroblasts after the induction of DNA damage. *Crbn* KO fibroblasts exhibited more severe MMP reduction than the WT fibroblasts, as indicated by tetramethylrhodamine methyl ester (TMRM) staining (Fig. 1c, d), which is in concert with the result obtained from PI staining. Furthermore, flow cytometry analyses showed that expression of CRBN protects cells from etoposide-induced apoptosis (Supplementary Figure S2). Taken together, our data indicate that CRBN exhibits protective roles in DNA damage-induced apoptosis.

### p53 is required for the regulation of CRBN on DNA damage-induced apoptosis

**Fig. 2:**
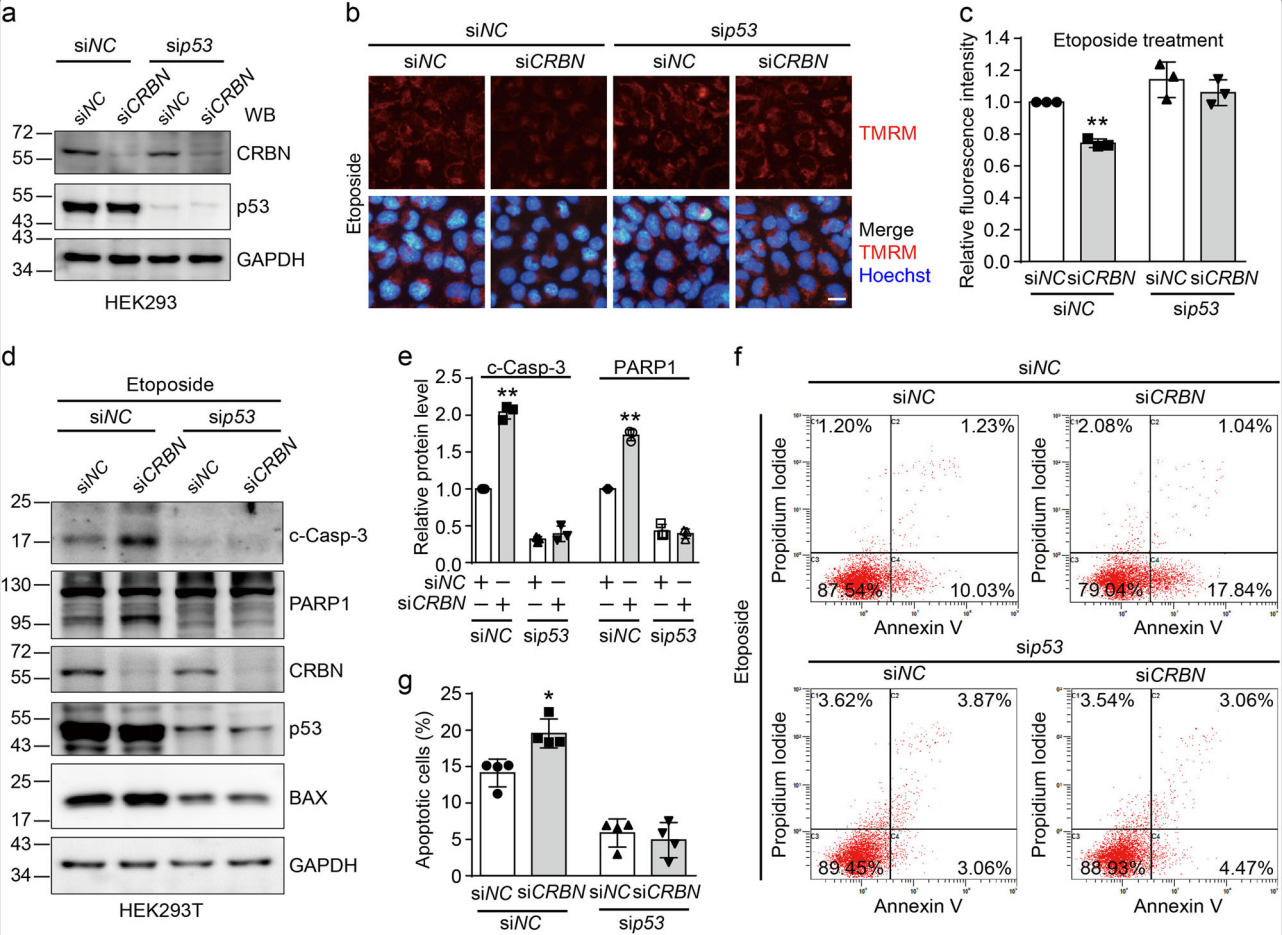
Cereblon (CRBN) inhibits etoposide-induced apoptosis in a p53-dependent manner. **a** Validation of knockdown efficiency for *CRBN* and *p53*. Human embryonic kidney (HEK) 293 cells were transfected with si*NC* or si*CRBN* along with si*NC* or si*p53* for 48 h, lysed, and the resulting cell lysates were subjected to immunoblotting analysis using the indicated antibodies. **b** *p53* knockdown attenuates the effect of CRBN on the mitochondrial membrane potential. HEK293 cells were transfected with si*NC* or si*CRBN* along with si*NC* or si*p53* for 40 h and then treated with etoposide (50 μM) for 8 h. Cells were stained with tetramethylrhodamine methyl ester (TMRM) and visualized under fluorescence microscope. Scale bar: 20 µm. **c** Quantitative data (mean ± SD) of **b** from three independent experiments. **P < 0.01, Student’s *t*-test. **d** *p53* knockdown prevents the cleavage of caspase-3 and poly(ADP-ribose) polymerase 1 (PARP1) induced by CRBN depletion upon etoposide treatment. HEK293T cells were transfected with si*NC* or si*CRBN* along with si*NC* or si*p53* for 24 h and then treated with etoposide (50 μM) for 48 h. Cells were lysed and cell lysates were subjected to immunoblotting analysis. **e** Quantitative data (mean ± SD) of **d** from three independent experiments. **P < 0.01, Student’s *t*-test. **f** *p53* knockdown eliminates the effect of *CRBN* knockdown on apoptosis induced by etoposide. HEK293T cells were treated as in **d** and subjected to flow cytometry analysis. **g** Quantitative data (mean ± SD) of **f** from three independent experiments. *P < 0.05, Student’s *t*-test

Considering the critical role of p53 in DNA damage-induced apoptosis^1^, we examined whether p53 is involved in the CRBN-mediated protection of DNA damage-induced apoptosis. As etoposide could activate the apoptotic mitochondrial pathway followed by activation of executioner caspase-3 (Fig. 1c)^41,42^, we then investigated the effects of knockdown of *CRBN* and *p53* by small interfering RNA (siRNA) on the MMP. Immunoblotting experiments confirmed the knockdown efficiency of *CRBN* and *p53* in human embryonic kidney (HEK) 293 cells (Fig. 2a). *CRBN* knockdown results in more severe reduction of MMP than the control knockdown, whereas further knocking down *p53* restores MMP in HEK293 cells when exposed to etoposide (Fig. 2b, c) although MMP is not changed in the absence of etoposide (Supplementary Figure S3a). We further found that *p53* knockdown inhibits the caspase-3 activation and PARP1 cleavage induced by etoposide in HEK293T cells (Fig. 2d, e and Supplementary Figure S3b). Although the protein level of BAX is decreased upon *p53* knockdown, CRBN does not affect the BAX protein level in the presence or absence of p53 (Fig. 2d, e). This result indicates that CRBN most probably does not regulate the p53 transcription activity. The flow cytometry analyses also demonstrated the importance of p53 in the CRBN-mediated regulation of etoposide-induced apoptosis (Fig. 2f, g, Supplementary Figures S3c, d).

### CRBN directly interacts with p53

**Fig. 3:**
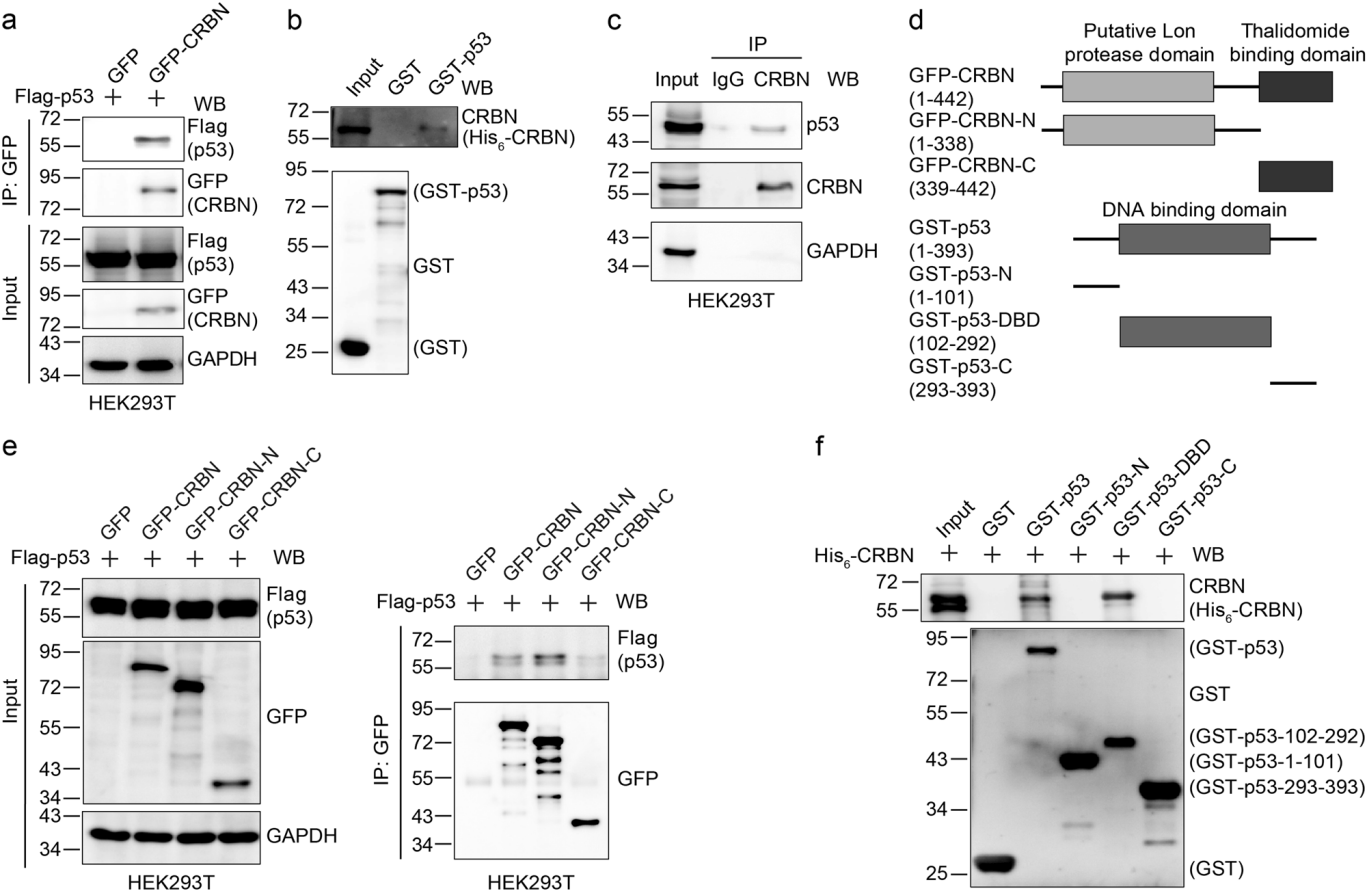
Cereblon (CRBN) interacts with p53. **a** CRBN immunoprecipitates p53 in ectopically expressed cells. Human embryonic kidney (HEK) 293T cells were co-transfected with green fluorescent protein (GFP) or GFP-CRBN and Flag-p53 for 48 h and the resulting cell lysates were subjected to immunoprecipitation. Cell lysates (input) and immunoprecipitates (IP) were immunoblotted with the indicated antibodies. **b** p53 directly interacts with CRBN. Glutathione *S*-transferase (GST) or GST-tagged p53 on glutathione agarose beads was incubated with His_6_-CRBN for 3 h at 4 °C. The beads were washed with phosphate-buffered saline (PBS) and bound proteins were eluted and analyzed by immunoblotting. **c** CRBN and p53 interact endogenously. HEK293T cell lysates were immunoprecipitated using anti-IgG or anti-CRBN antibody. Cell lysates and immunoprecipitates were subjected to immunoblotting analysis using the indicated antibodies. **d** The schematic representation of CRBN truncation mutants with GFP-tag at the N terminus and p53 truncation mutants with GST-tag at the N terminus. **e** p53 interacts with the CRBN N terminus. HEK293T cells were transfected with Flag-p53 and GFP, GFP-CRBN, GFP-CRBN-N (1–338), or GFP-CRBN-C (339–442), respectively, for 48 h. Cell lysates were immunoprecipitated using anti-GFP antibody. Cell lysates and immunoprecipitates were subjected to immunoblotting analysis using the indicated antibodies. **f** CRBN interacts with the p53 DNA-binding domain (DBD). GST, GST-tagged p53, or GST-tagged p53 mutants on glutathione agarose beads were incubated with His_6_-CRBN for 3 h at 4 °C. The beads were washed with PBS and bound proteins were eluted and analyzed by immunoblotting

Since our above experiments found that p53 is required for the CRBN-mediated regulation of etoposide-induced apoptosis, we then examined the possible interaction between CRBN and p53 by performing co-immunoprecipitation and in vitro glutathione *S*-transferase (GST)-pulldown experiments. We co-transfected Flag-p53 with green fluorescent protein (GFP) or GFP-CRBN into HEK293T cells and the cell lysates were subjected to immunoprecipitation 48 h after transfection. We found that GFP-CRBN but not GFP binds to Flag-p53 in HEK293T cells (Fig. 3a). The interaction between CRBN and p53 was further confirmed by performing a GST-pulldown assay (Fig. 3b), indicating their direct interaction. Co-immunoprecipitation of endogenous proteins in HEK293T cells and subsequent immunoblotting experiments revealed that CRBN interacts with p53 endogenously (Fig. 3c). To further narrow down the binding region between CRBN and p53, we constructed the CRBN and p53 truncation mutants (Fig. 3d) and performed protein interaction experiments. The results showed that the N terminus but not the C terminus of CRBN interacts with p53 (Fig. 3e), and CRBN binds to the DNA-binding domain (DBD) of p53 (Fig. 3f). Taken together, these data demonstrate that CRBN directly binds to p53.

CRBN is the primary target of a group of IMiDs^19^ including thalidomide, lenalidomide, and pomalidomide, which further promotes the interaction between CRBN and its substrates for their subsequent ubiquitination and degradation^26,27^. However, our experiments showed that lenalidomide and pomalidomide do not alter the interaction between CRBN and p53 (Supplementary Figure S4). This suggests that p53 might not be the ubiquitination substrate of the CRBN-associated E3 ligase. Alternatively, IMiDs might not affect the ubiquitination of p53, which is also consistent with the fact that p53 interacts with CRBN N terminus (Fig. 3e) while IMiDs interact with CRBN C terminus^19^ and the fact that IMiDs do not regulate the ubiquitination of a protein, which interacts with the CRBN N terminus^43^. Although two CRBN mutants C391R and R419X cause different degree of intellectual disability^33,35^, these mutations did not affect the interaction between CRBN and p53 (Supplementary Figure S5), presumably because the mutated region is far away from p53-binding domain. This result suggests that the learning and memory deficits in those patients might not be closely linked to the p53 functions.

### CRBN does not affect p53 transcription activity

**Fig. 4:**
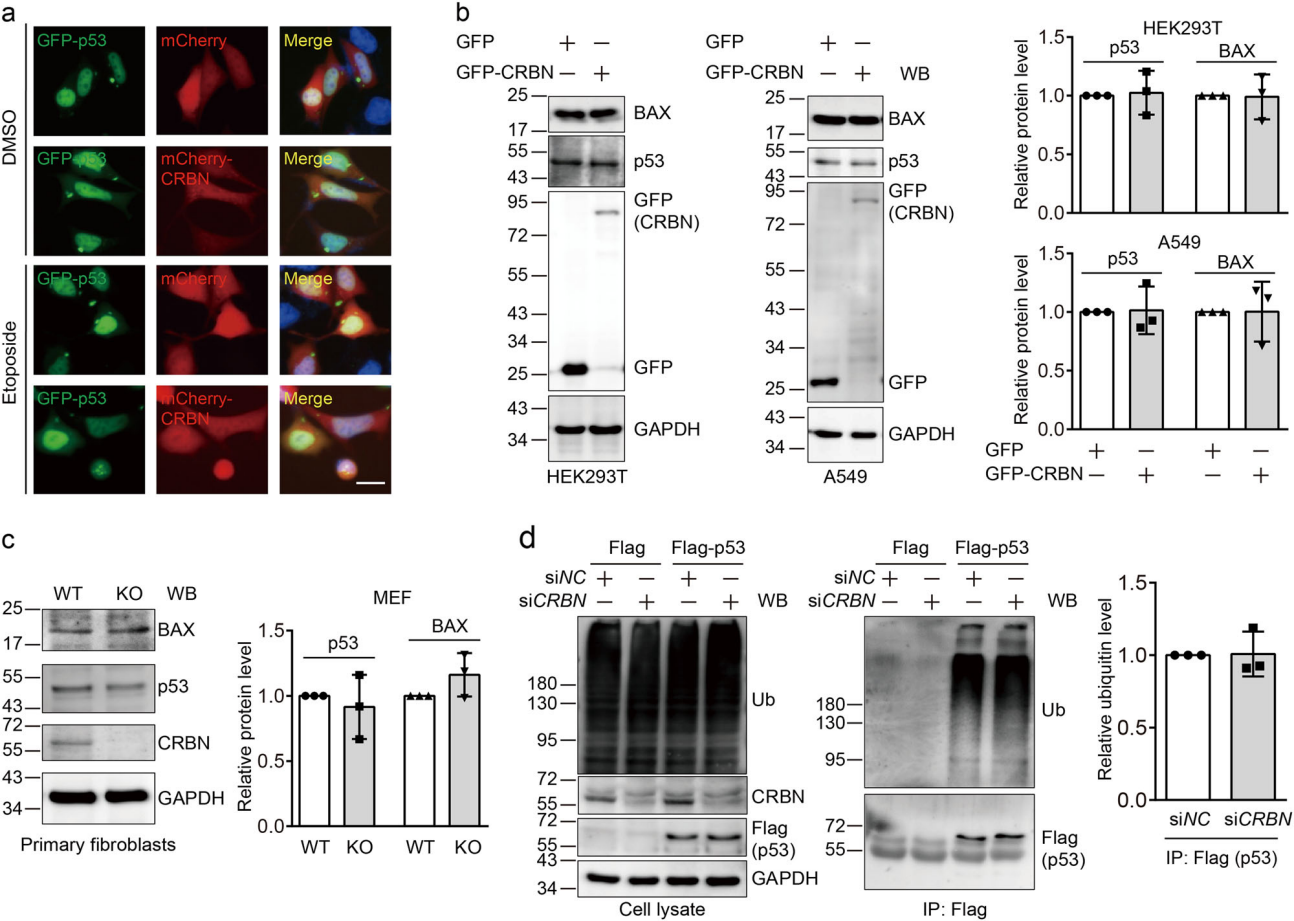
Cereblon (CRBN) does not change the subcellular distribution, protein level, and ubiquitination of p53. **a** CRBN does not affect p53 subcellular distribution. Human embryonic kidney (HEK) 293 cells were co-transfected with GFP-p53 and mCherry or mCherry-CRBN for 24 h and subcellular distribution of p53 was examined using immunofluorescence assay. Scale bar: 20 µm. **b** CRBN expression does not alter the protein level of p53 and BAX in HEK293T and A549 cells. HEK293T or A549 cells were transfected with GFP or GFP-CRBN for 48 h and the resulting cell lysates were subjected to immunoblotting analysis with the indicated antibodies. **c** *Crbn* deficiency does not influence the p53 and BAX protein level in primary fibroblasts. Primary fibroblasts from wild-type (WT) and *Crbn* knockout (KO) littermate mice at postnatal day 0–2 were cultured for 48 h, lysed, and the resulting cell lysates were subjected to immunoblotting analysis. **d** *CRBN* knockdown does not alter p53 ubiquitination. HEK293T cells were transfected with si*NC* or si*CRBN* for 24 h, again transfected with Flag or Flag-p53 for 48 h, and treated with MG132 (10 μM) for 6 h. Cells were lysed and the resulting cell lysates were immunoprecipitated using an anti-Flag antibody. The cell lysates and immunoprecipitates were immunoblotted with the indicated antibodies. Quantitative data (mean ± SD) in **b**–**d** were obtained from three independent experiments. Statistics using Student’s *t*-test indicated no statistical significance

Given the importance of subcellular distribution of p53 on its functions, we first investigated the possible influence of CRBN on the distribution of p53. To do so, we co-transfected GFP-p53 and mCherry-CRBN into HEK293 cells for immunofluorescence measurement. Our result showed that the nuclear localization of p53 is not influenced by CRBN under normal condition and etoposide-induced stress (Fig. 4a). In addition, two CRBN mutants do not affect the subcellular localization of p53 (Supplementary Figure S6). It has been discovered that p53 promotes cell death via its transcriptional upregulation of *BAX*, leading to the increased BAX protein level^5^. However, our experiments showed that the protein level of p53 and BAX is not altered upon expression of CRBN in HEK293T cells and A549 cells (Fig. 4b). Consistent results were observed in primary fibroblasts from WT and *Crbn* KO littermate mice (Fig. 4c). Although it has been found that CRBN could promote the ubiquitination of its binding partners such as CLC-1^21^ and c-Jun^25^, our experiments did not detect the change of p53 ubiquitination upon CRBN knockdown in HEK293T cells (Fig. 4d). Taken together, these data indicate that CRBN does not regulate the subcellular localization, ubiquitination, stability, and transcription activity of p53.

### CRBN affects the transcription-independent function of p53

**Fig. 5:**
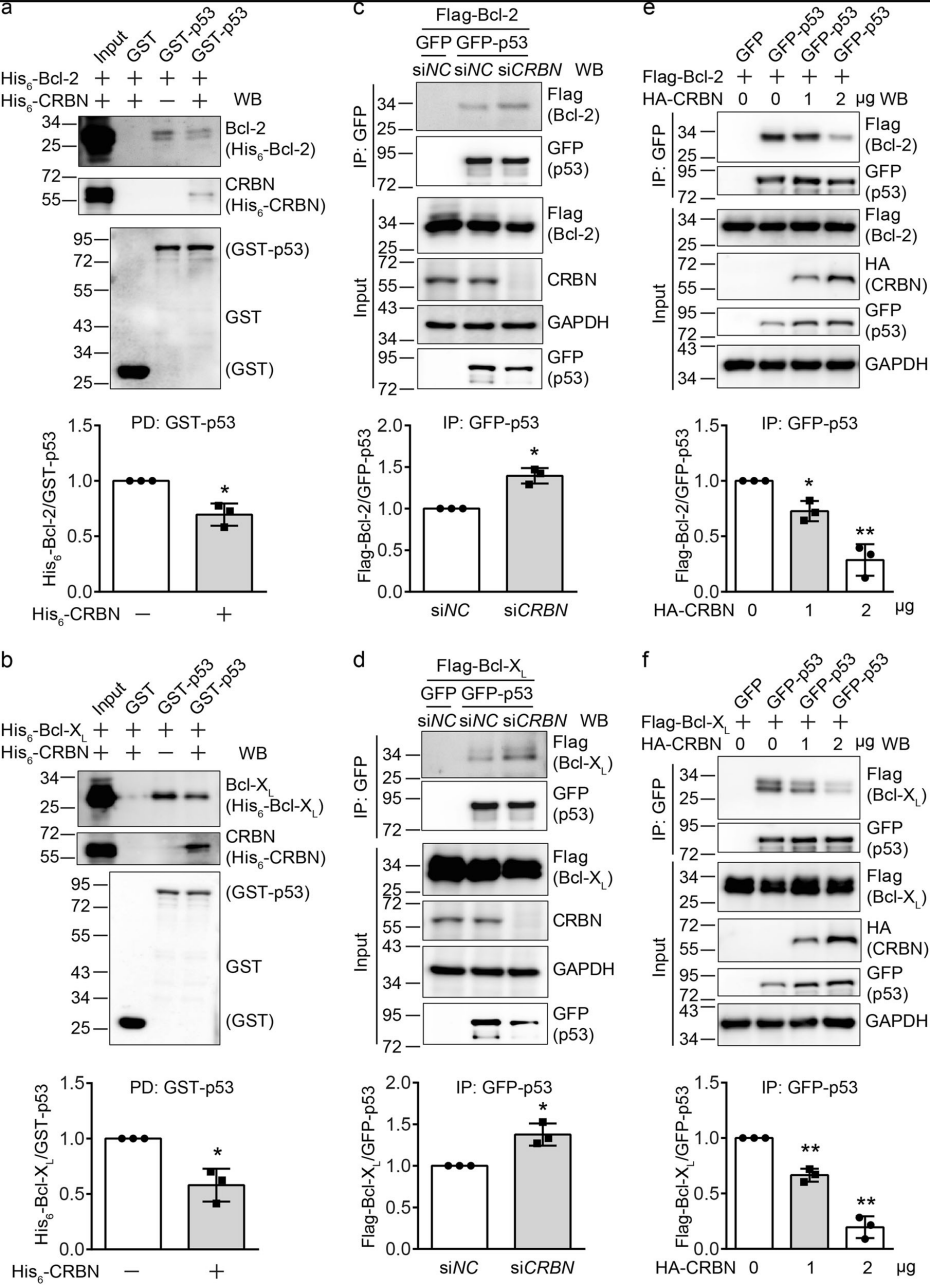
Cereblon (CRBN) attenuates the interaction between p53 and Bcl-2 or Bcl- X_L_. **a**, **b** CRBN reduces the interaction between p53 and Bcl-2 (**a**) or Bcl-X_L_ (**b**) in vitro. Glutathione agarose beads with purified glutathione *S*-transferase (GST) or GST-p53 were incubated with His_6_-Bcl-2 (**a**) or His_6_-Bcl-X_L_ (**b**) in the absence or presence of His_6_-CRBN at 4 °C for 3 h. The agarose beads were washed with phosphate-buffered saline and bound proteins were eluted and subjected to immunoblotting analysis using the indicated antibodies. **c**, **d** CRBN knockdown enhances the interaction between p53 and Bcl-2 (**c**) or Bcl-X_L_ (**d**). Human embryonic kidney (HEK) 293T cells were first transfected with si*NC* or si*CRBN* for 24 h and then co-transfected again with GFP or GFP-p53 and Flag-Bcl-2 (**c**) or Flag-Bcl-X_L_ (**d**) for 48 h. Cells were lysed and the resulting cell lysates were immunoprecipitated with an anti-GFP antibody. Cell lysates and immunoprecipitates were subjected to immunoblotting analysis using the indicated antibodies. **e**, **f** CRBN reduces the interaction between p53 and Bcl-2 (**e**) or Bcl-X_L_ (**f**) in HEK293T cells. Flag-Bcl-2 or Flag-Bcl-X_L_ was co-transfected into HEK293T cells in 6-cm plates with GFP or GFP-p53 in the absence or in the presence of increasing amounts of HA-CRBN (0, 1, and 2 μg). The cell lysates were immunoprecipitated with an anti-GFP antibody, and then the cell lysates and immunoprecipitates were subjected to immunoblotting analysis using the indicated antibodies. Quantitative data (mean ± SD) in **a**–**f** were from three independent experiments. Student’s *t*-test: *P < 0.05, **P < 0.01

It has been reported that p53 exhibits the transcription-independent function through its directly interaction with Bcl-2 and Bcl-X_L_^10,11^. Since CRBN binds to the p53 DBD (Fig. 3f), which is also the binding domain for Bcl-2 and Bcl-X_L_^10,14^, we further examined whether CRBN possibly modulates its transcription-independent function by altering the interaction between p53 and Bcl-2 and Bcl-X_L_. We purified GST-p53, His_6_-CRBN, His_6_-Bcl-2, and His_6_-Bcl-X_L_, which were individually expressed in *Escherichia coli*, and then performed GST-pulldown assay in vitro. The result indicates that p53 directly interacts with Bcl-2, but the interaction of p53 with Bcl-2 is decreased in the presence of CRBN (Fig. 5a). A similar result was also observed for Bcl-X_L_ (Fig. 5b). Furthermore, the interaction of p53 with Bcl-2 or Bcl-X_L_ is increased when we knocked down *CRBN* in HEK293T cells using the *CRBN*-specific siRNAs (Fig. 5c, d), and gradually decreased when we overexpressed increasing amount of CRBN in HEK293T cells (Fig. 5e, f). Together, our results demonstrated that CRBN affects the transcription-independent function of p53 by directly suppressing the interaction between p53 and Bcl-2/Bcl-X_L_.

### *Crbn* KO mice exhibit a higher mortality rate following etoposide challenge

**Fig. 6:**
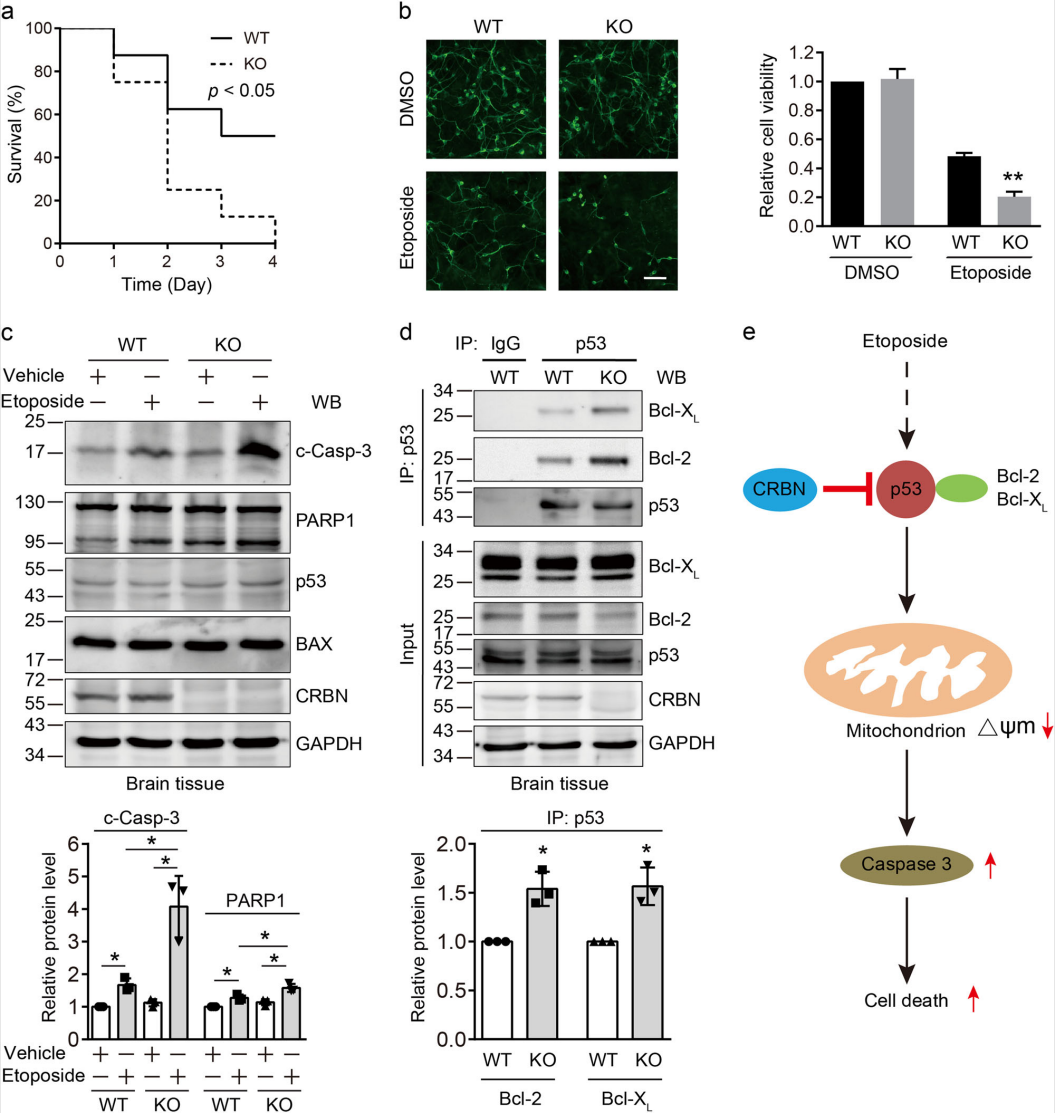
*Crbn* deficiency elevates the mortality rate of mice upon etoposide challenge. **a** *Crbn* deficiency promotes the death of mice after etoposide injection. Wild-type (WT) (n = 8) and *Crbn* knockout (KO) (*n* = 8) mice were intraperitoneally injected with 100 mg/kg etoposide. Mice were monitored for 4 days. *P < 0.05, log-rank (Mantel-cox) test. **b** *Crbn* KO reduces the viability of cortical neurons upon etoposide treatment. Primary cortical neurons from WT and *Crbn* KO littermate mice were treated with etoposide (5 μM) for 24 h and then subjected to immunofluorescence assay using the neuron-specific marker MAP2 to count the survival neurons. Scale bar: 50 µm. **c** *Crbn* KO promotes caspase-3 and poly(ADP-ribose) polymerase 1 (PARP1) cleavage in mouse brain tissues upon etoposide treatment. WT and *Crbn* KO littermate mice were subjected to intraperitoneal injection with 100 mg/kg etoposide or vehicle [30% propylene glycol, 5% Tween 80, and 65% D5W (5% dextrose in water)] for 24 h and then the brain tissues were dissected out and subjected to immunoblotting analysis using the indicated antibodies. **d** *Crbn* KO enhances the interaction between p53 and Bcl-2 or Bcl-X_L_ in brain tissues. WT and *Crbn* KO littermate mice were subjected to intraperitoneal injection with 100 mg/kg etoposide for 24 h and then the brain tissues were collected, lysed, and subjected to immunoprecipitation using an anti-IgG or anti-p53 antibody. The cell lysates and immunoprecipitates were immunoblotted with the indicated antibodies. Quantitative data (mean ± SD) in **b**–**d** were obtained from three independent experiments. Statistics: Student’s *t*-test, *P < 0.05, **P < 0.01. **e** Proposed model for the regulation of cereblon (CRBN) on the etoposide-induced apoptosis. p53 binds to Bcl-2 and Bcl-X_L_, which reduces mitochondrial membrane potential (Δψm) after etoposide treatment, leads to the enhanced cleavage of caspase-3 and PARP1, and results in apoptosis. CRBN attenuates the interaction between p53 and Bcl-2/Bcl-X_L_ by its competitive interaction with p53 and thus reduces the mitochondrion-induced cell death

Our results showed that CRBN affects the transcription-independent function of p53 and modulates the DNA damage-induced apoptosis. We then examined whether the mortality rate of mice is affected by *Crbn* KO upon etoposide challenge. The survival rate was monitored over 4 days after WT and *Crbn* KO mice were challenged with etoposide (100 mg/kg, intraperitoneal injection). All *Crbn* KO mice received etoposide (100 mg/kg) died within 4 days of injection whereas 50% of WT mice survived 4 days after injection (Fig. 6a), which is consistent with the previous study that the median lethal dose of etoposide for mice is about 100 mg/kg^44^. Because the central nervous system is susceptible to DNA damage-induced apoptosis^45,46^, we cultured cortical neurons obtained from WT and *Crbn* KO littermate mice and treated them with etoposide. The data showed that *Crbn* KO cortical neurons are more sensitive to etoposide than WT cortical neurons (Fig. 6b and Supplementary Figure S7). Immunoblotting of brain tissues showed that there is much more cleaved caspase-3 in *Crbn* KO mice than in WT mice following etoposide challenge (Fig. 6c). Protein interaction analyses using these brain lysates further showed that more Bcl-2 and Bcl-X_L_ bind to p53 in *Crbn* KO mice than in WT mice (Fig. 6d), confirming the physiological role of CRBN on the interaction between p53 and Bcl-2/Bcl-X_L_. Taken together, these data indicate that CRBN inhibits DNA damage-induced apoptosis by controlling the transcription-independent function of p53 (Fig. 6e).

## Discussion

It has been found that multiple CRL4 E3 ligase complexes regulate DNA damage-induced apoptosis^36–40^. The functions of one of their substrate receptors CRBN in DNA damage response remain elusive^37^ although this protein has been utilized for targeted protein degradation in potential cancer therapy. In this study, we found that primary fibroblasts and cortical neurons from *Crbn* KO mice are more sensitive to etoposide than those from their WT littermate mice (Figs. 1 and 6b), which suggests that CRBN protects DNA damage-induced apoptosis in vitro. In vivo experiments also showed that *Crbn* KO mice are more susceptible to etoposide challenge, evidenced by the fact that KO mice exhibit higher mortality rate than WT mice (Fig. 6a). Taken together, our results demonstrated that CRBN is involved in the regulation of DNA damage-induced apoptosis in vitro and in vivo.

p53 plays critical roles in DNA damage-induced apoptosis^1,2^. However, the molecular mechanisms by which p53 regulates DNA damage-induced apoptosis are not completely elucidated^42,47^. Several studies indicated that both the transcriptional function and transcription-independent function of p53 are involved in DNA damage-induced apoptosis^41,42,48,49^. It has been discovered that CRBN is required for the anti-myeloma activity of IMiDs^19,50^ but IMiDs cannot improve the overall survival of multiple myeloma patients with p53 deficiency^51^, indicating the possible molecular linkage between CRBN and p53. In this study, we demonstrated that the regulation of DNA damage-induced apoptosis by CRBN depends on p53 (Fig. 2). Although CRBN functions as a substrate receptor of the CRL4 E3 ligase and mediates the degradation of many substrates, it does not affect the ubiquitination and protein level of p53 and thus BAX protein level, indicating that CRBN does not regulate the ubiquitin-mediated degradation of p53 (Fig. 4) and its transcriptional function. Instead, the GST-pulldown and co-immunoprecipitation experiments discovered that CRBN expression decreases while *CRBN* knockdown enhances the interaction of p53 with Bcl-2 and Bcl-X_L_ through its competitive interaction with p53, indicating that CRBN modulates the transcription-independent function of p53 (Fig. 5). In short, this work revealed a novel mechanism for CRBN in the regulation of DNA damage-induced apoptosis. This provides another example where CRBN plays regulatory functions not associated with the enzymatic activity of CRL4^CRBN^ E3 ligase.

CRBN is the primary target of IMiDs and promotes the ubiquitination and degradation of two transcription factors IKZF1 and IKZF3 in the presence of IMiDs^19,26,27^. Through this mechanism, IMiDs exhibit their anti-myeloma activity. Although CRBN knockdown initially induces myeloma cell cytotoxicity^50,52^, CRBN protein level is decreased in IMiD-resistant myeloma cells^50,53^. Here we found that CRBN deficiency does not alter the apoptosis of primary fibroblasts and primary cortical neurons and the mortality rate of mice under normal condition although *Crbn*-deficient mice exhibit aberration on learning and memory^54^. However, under DNA damage stress, CRBN deficiency increases apoptosis of cell lines and primary cells.

Two CRBN mutants C391R and R419X have been discovered in children with different degree of intellectual disability^33,35^ although the exact molecular mechanism for the cause of this disease is unknown. It has been discovered that many proteins associated with intellectual disability are also involved in cellular response to DNA damage^55,56^. Despite the fact that the two ectopically expressed CRBN mutants bind to p53 to a degree similar to the WT CRBN, we cannot completely rule out the possible influence of p53 on the deficits of neuronal development and intellectual disability by CRBN mutation under stress since it has been shown that at least one of the mutants has reduced stability^34^. Autophagy is extensively involved in neuronal injury and death^57–59^. Although CRBN does not affect the autophagy under physiological condition^31^, it requires further investigation to determine whether CRBN modulates the p53-related autophagy in neurons upon DNA damage^60^.

In conclusion, we discovered a new function for CRBN in the regulation of DNA damage-induced apoptosis after etoposide and cisplatin treatment and revealed the underlying molecular mechanism. In this mechanism, CRBN does not regulate the ubiquitin-mediated degradation of p53 and the protein level of its downstream apoptotic regulator BAX. However, the interaction between CRBN and p53 attenuates the transcription-independent function of p53 by decreasing the interaction of p53 with Bcl-2 and Bcl-X_L_, thus protecting cells from DNA damage-induced apoptosis. This work would extend our understanding of the role of CRBN on DNA damage response and demonstrate the protective roles of CRBN under stress.

## Materials and methods

### Animals

*Crbn* KO mice were provided by Dr. Yong Cang at Zhejiang University (China) and bred in the animal facility at Soochow University. For in vivo drug treatment, etoposide was dissolved in a buffer containing 30% propylene glycol, 5% Tween 80, and 65% D5W (5% dextrose in water) and intraperitoneally administered to the WT and *Crbn* KO mice at a dose of 100 mg/kg. Mice were checked every 24 h to determine their survival. All animal study protocols were approved by the Institutional Animal Care and Use Committee of Soochow University.

### Plasmids

Plasmids expressing GFP-p53, GST-p53, Flag-p53, Flag-Bcl-X_L_, His_6_-Bcl-X_L_, Flag-Bcl-2, His_6_-Bcl-2, His_6_-CRBN, GFP-CRBN, GFP-CRBN-N (1–388), and GFP-CRBN-C (339–442) were obtained from previous work^31,61–63^. p53 cDNA was amplified from the Flag-p53 plasmid and subsequently inserted into PA-mCherry-C1 vector at *Xho*I/*Bam*HI sites to obtain mCherry-p53 plasmid. The primers used for PCR were CCGCTCGAGAAATGGAGGAGCCGCAGTC (sense) and CGCGGATCCTCAGTCTGAGTCAGG (antisense) from GeneWiz (Suzhou, Jiangsu, China).

### Cell culture and transfection

HEK293 and 293T cells and primary dermal fibroblasts were grown in Dulbecco’s modified Eagle’s medium (DMEM) (HyClone, Logan, UT, USA) containing 10% fetal bovine serum (FBS, Lonsera, Lonsa Science SRL, Uruguay), 100 units/mL penicillin, and 100 µg/mL streptomycin (Gibco, Grand Island, NY, USA). Lipofectamine 3000 (Invitrogen, Carlsbad, CA, USA) was used to transfect plasmids and RNAiMAX (Invitrogen) was used to transfect siRNAs in HEK293 and 293T cells. All siRNAs and a negative control (si*NC*: UUCUCCGAACGUGUCACGUdTdT) were purchased from GenePharma (Shanghai, China). The sequences were: si*p53*, sense: GACUCCAGUGGUAAUCUACU, antisense: AGUAGAUUACCACUGGAGUC; si*CRBN*, sense: CCCAGACACUGAAGAUGAAAU, antisense: AUUUCAUCUUCAGUGUCUGGG.

### Primary cultures of dermal fibroblasts and cortical neurons

Primary dermal fibroblasts were dissected from mice at postnatal day 0–2 and cultured according to a method described previously^31^. Briefly, head, tail, and limbs of mice were amputated with surgical scissors. Viscera were discarded and skin was put in pre-chilled Hanks balanced salt solution. Tissue was cut into ∼1 mm pieces and digested at 37 °C for 20 min with 0.25% trypsin solution (Gibco). Cells were collected after centrifugation and resuspended in DMEM containing 10% FBS, 100 units/mL penicillin, and 100 µg/mL streptomycin.

Cortical tissues from neonatal mice at postnatal day 0–2 were dissected and digested with 0.25% trypsin solution to obtain individual cells. Cells were plated at 800,000 cells/well in 24-well plates coated with poly-d-lysine (Sigma, St. Louis, MO, USA) with neurobasal plating medium containing 2% FBS, 2% B27 supplements (Gibco), and 2 mM l-glutamine (Gibco). Neurobasal culture medium containing 2% B27 supplements and 2 mM l-glutamine was used to replace half of the medium every other day.

### PI and TMRM staining

Primary dermal fibroblasts, primary cortical neurons, and HEK293 cells were cultured and treated with dimethyl sulfoxide, etoposide (Selleck, Houston, TX, USA), or cisplatin (Selleck). Cells were incubated with Hoechst (Sigma) and PI (Beyotime, Haimen, Jiangsu, China) for 5 min, washed with phosphate-buffered saline (PBS), and detected under a fluorescence microscope.

TMRM staining was carried out according to a previous procedure to measure the mitochondrial membrane potential^64^. Briefly, cells were treated with 100 nM TMRM (Sigma) at 37 °C for 15 min, washed with PBS, and detected under a fluorescence microscope.

### Immunoprecipitation

HEK293T cells or tissues for mice were lysed with RIPA lysis buffer [25 mM Tris-HCl (pH 7.6), 150 mM NaCl, 1% NP-40, 1% sodium deoxycholate, and protease inhibitor cocktail (Roche, Basel, Switzerland)]. Immunoprecipitation was carried out using a previous method^65^. The rabbit polyclonal anti-GFP antibodies, rabbit or mouse IgG (Beyotime), or mouse monoclonal anti-p53 antibodies (Santa Cruz Biotechnology, Santa Cruz, CA, USA) were incubated with protein G sepharose beads (Roche) for 3–6 h and then incubated with cell lysates for 8–12 h at 4 °C. The protein G sepharose beads were washed six times with RIPA lysis buffer and subjected to heating with 2× SDS sample loading buffer. The eluted proteins were immunoblotted with the indicated antibodies.

### In vitro pulldown assay

GST-pulldown assay was performed according to a method described previously^66^. All tagged proteins used here were expressed in *E. coli*. Briefly, 30 µL of glutathione agarose beads (Amersham Pharmacia Biotech, Piscataway, NJ, USA) were incubated with GST or GST-tagged proteins for 30–60 min at 4 °C and washed three times with pre-chilled PBS. Beads were further incubated with His_6_-tagged CRBN alone or with Bcl-2 or Bcl-X_L_ for 3 h at 4 °C and again washed five times with pre-chilled PBS. GST-tagged proteins were eluted with 2× SDS sample loading buffer for immunoblotting analysis.

### Immunoblotting

Proteins were separated by SDS-polyacrylamide gel electrophoresis, transferred to polyvinylidene difluoride membrane (Millipore, Bedford, MA, USA), and immunoblotted according to a previous procedure^67^. The following primary antibodies were used for immunoblotting: Flag (Sigma), GFP (Santa Cruz Biotechnology), GAPDH (HuaAn Biotechnology, Hangzhou, Zhejiang, China), rabbit Bcl-2 (Epitomics, Burlingame, CA, USA), rabbit Bcl-X_L_ (Cell Signaling Technology, Danvers, MA, USA), mouse CRBN (from Xiu-Bao Chang, Mayo Clinic College of Medicine, USA), rabbit CRBN (ProteinTech, Rosemont, IL, USA), and anti-mouse IgG-horseradish peroxidase (HRP) or anti-rabbit IgG-HRP sheep antibodies (Amersham Pharmacia Biotech). Proteins were visualized using western blotting chemiluminescent horseradish peroxidase substrate (NCM Biotech, Suzhou, Jiangsu, China).

### Immunocytochemistry assay

First, primary cortical neurons or HEK293 cells were washed twice with PBS and fixed with 4% paraformaldehyde for 5–10 min at room temperature. Second, cells were permeabilized with 0.25% Triton X-100 at room temperature for 5 min and blocked with 5% FBS at room temperature for 1 h. Then, neuronal cells were incubated with microtubule-associated protein 2 antibody (Santa Cruz Biotechnology) at room temperature for 2–3 h and incubated with the Alexa Fluor 488 (green)-conjugated fluorescent secondary antibodies (Invitrogen) for 1 h. The cells were stained with Hoechst and visualized under an inverted microscope (IX71, Olympus, Tokyo, Japan).

### Data analysis

Photoshop 7.0 (Adobe Systems, San Jose, CA, USA) was used to analyze the densitometry of fluorescence and chemiluminescence from three independent experiments and the final data were analyzed using GraphPad Prism (GraphPad Software, La Jolla, CA, USA). *P*-values were calculated using Student’s *t*-test.

## Supplementary information

Supplemental material
