# Supplementary material for: Cereblon attenuates DNA damage-induced apoptosis by regulating the transcription-independent function of p53
Source: Cell Death Dis. 2019 Jan 25;10(2):69. doi: 10.1038/s41419-019-1317-7 (PMC6347596; doi:10.1038/s41419-019-1317-7)
Supplement: Supplementary file 1 — Supplemental material [file 41419_2019_1317_MOESM1_ESM.doc]

Supplementary Information

**Figure S1. Validation of wild-type (WT) and *Crbn* knockout (KO) mice.** (a) Genotype of WT and *Crbn* KO mice. Tails from mice (postnatal day 0-2) were cut and subjected to PCR genotyping using the following primers. *Crbn* forward: CAGTCAGATGGGTAAGGAGCA, reverse: AAGCAGCTCCGTAATGCTG; *Gapdh* forward: GGTGAAGGTCGGTGTGAACG, reverse: TTACGGGATGGGTCTGAACG. (b) Primary fibroblasts from WT and *Crbn* KO littermate mice were subjected to immunoblotting analysis using the indicated antibodies.

**Figure S2. CRBN protects cells against etoposide-induced apoptosis.** HEK293T cells were transfected with pcDNA3.1 or HA-CRBN for 24 h and treated with DMSO or etoposide (50 µM) for 48 h. (a) DMSO-treated cells were harvested, lysed, and the resulting cell lysates were subjected to Western blotting analysis to confirm protein expression. (b) Cells from all four samples were collected and analyzed by flow cytometry. (c) Quantitative data (mean ± SD) of (b) were from three independent biological experiments. *: *P* < 0.05, Student’s *t*-test.

**Figure S3. CRBN inhibits etoposide-induced apoptosis in a p53-dependent manner.** (a) HEK293 cells were transfected with si*NC* or si*CRBN* along with si*NC* or si*p53* using RNAiMAX transfection reagent for 40 h and then treated with DMSO for 8 h. Cells were stained with TMRM and examined under a fluorescence microscope to measure the mitochondrial membrane potential. Scale bar: 20 µm. Quantification (mean ± SD) was obtained from three independent biological experiments. (b) HEK293T cells were transfected with si*NC* or si*CRBN* along with si*NC* or si*p53* for 24 h and treated with DMSO for 48 h. Cells were then subjected to immunoblotting analysis using the indicated antibodies. (c) HEK293T cells were treated as in (b) and subjected to flow cytometry analysis. (d) Quantitative data (mean ± SD) of (c) were from three independent biological experiments.

**Figure S4. Immunomodulatory drugs (IMiDs) lenalidomide and pomalidomide do not affect the interaction between CRBN and p53.** HEK293T cells were transfected with GFP or GFP-CRBN along with Flag-p53 for 24 h and then treated with IMiDs (10 µM lenalidomide or 2 µM pomalidomide) for 24 h. Cells were harvested, lysed, and the resulting cell lysates were immunoprecipitated using anti-GFP antibody. Cell lysates and immunoprecipitates were subjected to immunoblotting analysis using the indicated antibodies.

**Figure S5. CRBN mutants associated with intellectual disability do not affect the interaction between CRBN and p53.** HEK293T cells were transfected with GFP, GFP-CRBN, GFP-CRBN (C391R), or GFP-CRBN (R419X) along with Flag-p53 for 48 h. Cells were harvested and the resulting cell lysates were immunoprecipitated using anti-GFP antibody. The cell lysates and immunoprecipitates were subjected to immunoblotting analysis using the indicated antibodies.

**Figure S6. CRBN mutants do not affect the subcellular distribution of p53.** HEK293 cells were transfected with GFP, GFP-CRBN, GFP-CRBN (C391R), or GFP-CRBN (R419X) along with mCherry-p53 for 24 h and examined under Olympus IX71 inverted optical microscope. Scale bar: 20 µm.

**Figure S7. *CRBN* KO enhances the etoposide-induced apoptosis in primary cortical neurons.** Primary cortical neurons from WT and *Crbn* KO littermate mice were treated with DMSO or etoposide (5 μM) for 24 h and then subjected to immunofluorescence staining using PI and Hoechst for counting of PI positive cells. Scale bar: 20 µm. Quantification was obtained from three independent biological experiments (mean ± SD). **: *P* < 0.01 against the sample from WT mice treated with etoposide, Student’s *t*-test.
